# 

**Published:** 1843-12

**Authors:** 


### WESTERN JOURNAL

OF

### MEDICINE AND SURGERY.

### EDITORS.

---

DANIEL DRAKE, M. D.,

PROFESSOR OF PATHOLOGICAL ANATOMY AND CLINICAL MEDICINE IN THE MEDICAL INSTITUTE OF LOUISVILLE:

LUNSFORD P. YANDELL, M. D.,

PROFESSOR OF CHEMISTRY AND PHARMACY IN THE SAME:

THOMAS W. COLESCOTT, M. D.

### INDEX TO VOL. VIII.

A
Abdominal neuralgia, 376.
Accelerating labor, new mode of, 370.
Account of the Lane Seminary fever, 322.
Advantages of cultivating medical physiognomy, 66.
Advantage of marriage, 471.
Ammonia in delirium tremens, 66.
Anatomy, lectures on, 80.
Anatomy, morbid, contributions to, 241.
Aneurism of common carotid, 161.
Antidote for bichloride of mercury, 232.
Anus, artificial, nature and treatment of, 1.
Arsenious acid detected in the liver, 220.
Artificial fountains, 73.
Assafœtida in hooping-cough, 71.
Astringent preparation, 234.
Asylum, lunatic, reports on, 114.
Avent's case of intermittent fever, 101.

B
Barbour on neuralgia, 401.
Bichloride of mercury, antidote for, 232.
Bird on abdominal neuralgia, 376.
Black cataract, 310.
Black tongue, Bennet on, 110.
Borlan on influenza, 274.
Botanical labors of Mutis, 232.
Brain, effusion in, 70.
Bridges' edition of Graham's chemistry, 396.
Buchanan's case of cancer, 81.

C
Caldwell on animal chemistry, 395, 430.
Cancer, Buchanan's case of, 81.
" frequency of, 306.
Carroll on the Lane Seminary fever, 322.
Cases in surgery, by Dr. Esselman, 88.
Cataract, Rosas' operation for, 304.
" black, 310.
Cerebro-spinal membranes, suppuration of, 130.
Chancre, treatment of, 383.
Characters, &c., of certain morbid growths, 367.
Cholera, treatment of, 390.
Clinical report on pneumonia in children, 184.
Club-foot, Hensley's operation for, 397.
Common carotid artery, aneurism of, 161.
Contact of primitive and tertiary rocks, 76.
Continental treatment of neuralgia, 388
Contributions to morbid anatomy, 241.
Cooper, Sir Astley, 462.
Curiosity in obstetric physiology, 147.
Curvature of the spine, 314.

D
Dawson on remitting fever, 418.
Death of Dr. Harlan, 400.
" of Judge Rowan, 159.
Delirium tremens treated by ammonia, 66.
Dental art, Maury's treatise on, 55.
Departure and acknowledgments, 78.
Diagnosis between epididymitis and orchitis, 471.
Difference of respiratory movements according to age, 385.
Dislocation into ischiatic notch, new sign of, 311.
Dislocation of semilunar cartilage, 470.
Doctors, old, in the south, 77.
Dowler's contributions to morbid anatomy, 241.
Dupierris on organic strictures of the urethre, 48.
Dura mater, osseous deposite in, 97.

E
Edinburgh professors, 233.
Effusion into the brain, 70.
Electro-magnetism in opium-poisoning, 129.
Emmenagogue medicines, 300.
Esselman's cases in surgery, 88.

Evanson and Maunsell on diseases of children, 55.
Experiments in homeopathy, by Andral, 368.
Eye, examination of, in disease, 372.
F
Ferruginated pill of mercury, 150.
Fever at Lane Seminary in 1842, 322.
Foreign bodies in joints, removal of, 266
Fountains, artificial, 73.
Fracture of the pelvis, &c., 130.
Free-and-easy style, 301.
Frequency of cancer, 306.
G
Galt's Practical Medicine, 476.
Gallic acid in menorrhagia, 394.
Goitre, 236.
Goyrand on removal of foreign bodies from joints, 266.
Graham's chemistry, 396.
Gross' monograph on artificial anus, 1, 161.
Gun-shot wound, &c. 126.
H
Harlan, Dr., death of, 400.
Headache, nervous, 390.
Health of the south, 319, 398.
Heart, functional disease of, &c., 104.
Hernia, strangulated, treated by exhaustion through elastic tube, 68.
Herpetic pruritus, 393.
Hodgkin on the characters of certain morbid growths, 367.
Homœopathic experiments, 368.
Hooping-cough, treated by assafœtida, 71.
Horse, normal proportions of, 388.
I
Influenza, 153, 159.
Intemperance, return of, 237.
Intermittent from birth, 101.
Intestine, strangulated from rotation of sigmoid flexure, 133.
Ioduretted syrup of sarsaparilla, 389.
Ipecacuhana, singular effects of, 95.
Iris, indications afforded by, in cerebral lesion. 151.
Ischiatic notch, sign of dislocation into, 311.
J
Jails, medical police of, 238.
Johnston's manual of chemistry, 400.
Joints, removal of foreign bodies from, 266.
K
Kidney, moveable, cases of, 313.
L
Labor, Staniland's method of accelerating, 370.
Lactation, new limits for, 311.
Lane seminary, Carroll on fever of, 322.
Lateral curvature of the spine, 314.
Lead in maccouba-snuff, poisoning by, 391.
Lectures on anatomy and operative surgery, 80.
Legalized quackery, 76.
Lithotrity, McDowell's case of, 364.
M
Maccouba-snuff, adulterated with lead, 391.
Maunsell and Evanson on children, 55.
McCormac on prolapsus ani, 231.
McDowell's operation of lithotrity, 364.
Medical Institute of Louisville, 400.
Medical police of jails, 238.
Medical physiognomy, advantage of cultivating it, 66.
Medical profession, 381.
Medical society of Tennessee, 78.
" " of Nashville, 79.
Medical writers good practitioners, 139.
Memoir on strictures of the urethra, 48.
Menorhagia, treated by gallic acid, 394.
Mesmerism, 76, 156.
Minorca, medical faculty of, 290.
Minor surgery, Smith's, 320.
Mississippi, voyage up, 153.
Molasses and water for burns, 390.
Monograph on artificial anus, 1.
Morbid anatomy, contrtbutions to, 241.
Moveable kidney, 313.
Mutis, botanical labors of, 232.
N
Nature and treatment of artificial anus, 1.
Necrosis of the skull, 296.
Neuralgia, abdominal, depending on uterine irritation, 376.
Neuralgia, Barbour on pathology of, 401.
Neuralgia, continental treatment of, 388.
Neuromatous tumors in stumps, structure of, 141.
New astringent preparation, 234.
New limits for the period of lactation, 311.
New method of removing foreign bodies from joints, 266.
New mode of accelerating labor, 370.
New preservative for animal substances, 390.
Normal proportions of the horse, 388.
O
Observations on some popular remedies of German practitioners, 57.
Obstetrical auscultation, 317.
Old doctors, 77.
Onyxis, 231.
Operation for cataract, Rosas', 304.
" for club-foot, 397.

Operation of lithotrity, McDowell's, 364.
Opium-poisoning, treated by electro-magnetism, 129.
Organic strictures of the urethra, 48.
Osseous deposit in the dura mater, 97.
P
Pelvis, fracture of, &c., 130.
Pennsylvania medical college, 319.
Physiognomy, medical, cultivation of, 66.
Pneumonia of children, by Dr. West, 184.
Pneumonia, treatment of, 142.
Poisoning by arsenious acid, detected in the liver, 220.
Poisoning by lead in maccouba-snuff, 391.
Popular remedies of German practitioners, 57.
Posey county, Indiana, diseases of, 170.
Practical treatise on the diseases of children, 55.
Primitive and tertiary rocks, contact of, 76.
Professors at Edinburgh, 233.
Progress of mesmerism, 156.
Prolapsus ani, 231.
Proportions of the horse, 388.
Pruritus of the vulva, 393.
" herpetic, 393.
Q
Quackery, legalized, in Alabama, 76.
Quinine in traumatic fever, 312.
R
Remitting fever, Dawson on, 418.
Remedies of German practitioners, 57.
Researches on the frequency of cancer, 306.
Respiratory movements at different ages, 385.
Return of intemperance, 237.
Robson on diseases of the Wabash Valley, 170.
Rocks, primitive and tertiary, contact of, 76.
Rosas' operation for cataract, 304.
Rotation of the sigmoid flexure—strangulation, 133.
S
Sale of secret remedies abroad, 467.
Sarsaparilla, ioduretted syrup of, 389.
Semilunar cartilage, dislocation of, 470.
Sigmoid flexure, rotation of—strangulation, 133.
Skull, necrosis of, 296.
Smith's minor surgery, 320.
Snuff adulterated with lead, 391.
South, health of, 319, 398.
Spine, lateral curvature of, 314.
Spinal accessory nerve, anatomy and phisyology of, 471.
Staniland's mode of accelerating labor, 370.
Strangulated hernia, treated by exhaustion through the elastic tube, 68.
Strictures of the urethra, memoir on, 48.
Structure of neuromatous tumors in stumps, 141.
Structural peculiarities of a group of morbid growths, in which cancerous affections are involved, 367.
Structure of the uterus, 149.
Surgery, operative, in the south-west, 77.
Syphilis, lecture on, 143.
T
Taraxacum, liquor of, 71.
Thou atomy thou, 476.
Traumatic fever, use of quinine in, 312.
Travelling editorials, 153, 236, 240.
Treacle and water for burns, 390.
Treatise on the dental art, 55.
Typhus abdominalis, 132.
U
Urethra, memoir on strictures of, 48.
Uterine irritation causing neuralgia, 376.
Uterus, structure of, 149.
V
Van Arsdale on obstetric auscultation, 317.
Varicose veins, treatment of, 393.
Voyage up the Mississippi, 153.
Vulva, pruritus of, 393.
W
Wabash valley, diseases of, 190.
Wendel on aneurism of the common carotid artery, 161.
Wenzel the oculist, 313.
Western Journal, 473.
West on the pneumonia of children, 184.
White county, Illinois, fever of, 170.
Worari poison and its effects, 468.
Wounds of the intestines and artificial anus, 160.
Y
Yellow fever in Pittsburg 156.

